# Analysis of adverse event reporting with casimersen: a pharmacovigilance study based on the United States food and drug administration adverse event reporting system database

**DOI:** 10.1007/s11096-026-02103-5

**Published:** 2026-02-26

**Authors:** Zhenghua Hao, Kaiyao Jiang, Junfeng Zhang, Linmei Zhao

**Affiliations:** 1 Department of Pharmacy, Second Hospital of Shanxi Medical University, No. 382, Wuyi Road, Taiyuan, 030001 China; 2 School of Pharmacy, Shanxi Medical University, Taiyuan, China; 3 The Seventh Clinical Medical School of Shanxi Medical University, Linfen, China

**Keywords:** Adverse events, Antisense oligonucleotides, Casimersen, Disproportionality analysis, FAERS, Real-world evidence

## Abstract

**Introduction:**

Casimersen is an antisense oligonucleotide used to treat patients with Duchenne muscular dystrophy (DMD), with mutations amenable to exon 45 skipping. However, real-world safety data are limited.

**Aim:**

This study used the Food and Drug Administration Adverse Event Reporting System (FAERS) database to describe post-marketing adverse event reporting patterns associated with casimersen, identify disproportionality signals at the preferred term level, and characterize their onset patterns and affected organ systems.

**Method:**

FAERS reports from 2004 to 2024 involving casimersen were extracted, deduplicated, and coded using the Medical Dictionary for Regulatory Activities (MedDRA). Disproportionality analyses were performed using four validated algorithms: Reporting Odds Ratio (ROR), Proportional Reporting Ratio (PRR), Bayesian Confidence Propagation Neural Network (BCPNN) and Empirical Bayesian Geometric Mean (EBGM). Signals that met all four criteria were considered statistically significant. Time-to-onset and subgroup analyses according to age and sex were also performed.

**Results:**

Among 21,964,449 FAERS reports, 598 listed casimersen as the primary suspect, predominantly in males (98.5%) and patients aged < 18 years (62.0%). The median time to AE onset was 253 days (range, 101–490 days). Twenty-one System Organ Classes (SOCs) were implicated, including injury, poisoning, procedural complications (n = 377), vascular disorders (n = 85), product issues (n = 80), and social circumstances (n = 13). Using all four algorithms, 30 significantly preferred terms (PTs) were identified, encompassing heterogeneous reporting categories, including clinically oriented terms as well as administration-related, medication use, and non-specific descriptors, such as product dose omission, poor venous access, proteinuria, hematuria, chromaturia, underdose, illness, and infusion-site extravasation.

**Conclusion:**

This study characterized post-marketing adverse event reporting patterns associated with casimersen using FAERS data. By summarizing the preferred term–level reporting distributions, affected organ system categories, and time-to-onset characteristics, the findings provide a descriptive overview of real-world reporting patterns following casimersen use. These results may inform post-marketing pharmacovigilance activities and support hypothesis generation in future studies.

**Supplementary Information:**

The online version contains supplementary material available at 10.1007/s11096-026-02103-5.

## Impact statements

- The reporting of renal-related preferred terms in post-marketing FAERS data highlights the importance of maintaining awareness of renal safety considerations when casimersen is used in routine practice, which is consistent with existing regulatory labeling and prior clinical experience.
- Reports involving infusion-site extravasation and venous access–related preferred terms highlight the procedural and administration-related challenges associated with repeated intravenous therapy in patients with DMD and may provide contextual information for clinical teams involved in treatment delivery.
- Preferred terms related to dose omission or underdose suggest that adherence- and process-related factors may be reflected in spontaneous reporting data, highlighting the potential value of multidisciplinary support and patient–caregiver communication in long-term treatment settings.

## Introduction

Duchenne muscular dystrophy (DMD) is a rare X-linked recessive neuromuscular disorder that primarily affects males, with an estimated prevalence of approximately 1 in every 4,000–5,000 male births. The disease arises from various pathogenic mutations, including exon deletions, nonsense mutations, insertions or deletions within exons, exon duplications, splice-site alterations, and intronic rearrangements. These genetic defects result in either the complete absence or malfunction of dystrophin, a critical cytoskeletal protein that stabilizes muscle fibers during contraction. The lack of functional dystrophin renders muscle fibers vulnerable to mechanical injury and progressive degeneration, ultimately leading to muscle weakness, loss of ambulation, and premature death, typically due to cardiorespiratory failure in the third decade of life [1].

Cognitive impairment, behavioral challenges, and reduced bone density with increased fracture risk are also commonly reported in patients [2–4]. Progressive muscle wasting and fibrosis may lead to joint contractures, scoliosis, and respiratory or cardiac complications, further exacerbating morbidity and mortality [5].

Antisense oligonucleotides (ASOs) have emerged as a promising therapeutic strategy for DMD. These single-stranded nucleic acid molecules modulate pre-mRNA splicing by binding to complementary mRNA sequences, thereby enabling the production of truncated, but functional dystrophin proteins. In 2021, the United States Food and Drug Administration (FDA) approved casimersen (Ammondys 45™), an exon 45–skipping ASO designed for patients with DMD gene mutations amenable to exon 45 skipping. Casimersen binds to exon 45 of dystrophin pre-mRNA, promoting its exclusion during splicing and facilitating the synthesis of a partially functional dystrophin protein [6, 7].

The drug demonstrated predictable pharmacokinetics with a steady-state plasma clearance of approximately 180 mL/h/kg and an elimination half-life of 3.5 h. More than 90% of casimersen is excreted unchanged in the urine, indicating that renal clearance is the principal elimination pathway [8]. Accordingly, renal function has been the focus of safety evaluations, particularly in patients with pre-existing kidney impairment. Clinical trials have primarily reported mild to moderate adverse events (AEs), including upper respiratory tract infection, headache, cough, and fever [9]. However, preclinical studies have indicated possible renal tubular toxicity, leading to FDA class warnings for nephrotoxicity across all ASO therapies.

Despite these insights, post-marketing safety information regarding casimersen remains limited, particularly with respect to spontaneously reported AEs in routine clinical practice. The FDA Adverse Event Reporting System (FAERS), the world’s largest open pharmacovigilance database, provides an important source of post-marketing safety data that can complement evidence from clinical trials by characterizing real-world reporting patterns [10]. Although FAERS-based investigations have been widely applied to explore AE reporting for various biological and small-molecule agents, published analyses focusing on casimersen remain scarce. Within this context, the present study used FAERS data to describe post-marketing AE reporting patterns associated with casimersen and to explore potential signals warranting further evaluation in future studies**.** FAERS-based analyses are derived from spontaneously reported Medical Dictionary for Regulatory Activities (MedDRA) preferred terms, which represent heterogeneous reporting categories encompassing clinically oriented events, medication-use processes, administration-related issues, and non-specific descriptors.

### Aim

This study aimed to describe post-marketing adverse event reporting patterns at the preferred term (PT) level of casimersen using the FAERS database by detecting disproportionality signals, characterizing affected organ systems and PTs, and analyzing time-to-onset and demographic subgroups to support post-marketing pharmacovigilance and hypothesis generation for further evaluation.

## Method

### Study design

This exploratory pharmacovigilance study assessed post-marketing AE reporting patterns associated with casimersen using the FAERS database. The analysis was hypothesis generating and intended to identify disproportionality signals rather than infer causal relationships.

### Data source

Data were retrieved from the FAERS database, which comprises spontaneous AE and medication error reports submitted by healthcare professionals, consumers, and manufacturers. Reports covering the period from the first quarter of 2004 to the third quarter of 2024 were extracted from the DRUG, REAC, and DEMO datasets. All cases listing casimersen as the primary suspected drug were included, while those identifying it as a concomitant or secondary suspected drug were excluded to ensure the specificity of the association.

### Data management and standardization

Duplicate reports were identified and removed using the FDA deduplication protocol [11]. For reports sharing identical CASEIDs, only the entry with the most recent FDA receipt date (FDA_DT) was retained. When both CASEID and FDA_DT were identical, the report with the highest PRIMARYID score was retained.

AEs were coded according to the MedDRA version 26.1, allowing classification of both System Organ Class (SOC) and PT levels [12]. Analyses were conducted at the MedDRA PT level, consistent with the structure of spontaneous reporting in FAERS, which allows systematic identification of disproportionality patterns, but may be limited in clinical interpretability for conceptually broad or process-related terms. Records with implausible or missing demographic information (e.g., age < 0 or > 120 years or unspecified sex) were excluded from descriptive analyses. Data were processed using Microsoft Excel Professional Plus 2013 (Microsoft Corp., Redmond, WA, USA).

### Subgroup and time-to-onset analysis

For subgroup evaluation, reports were stratified by age (< 18 vs. ≥ 18 years) and sex to explore demographic differences in the reported PT patterns. For time-to-onset analysis, the interval between the start date of casimersen therapy and the onset date of the reported AE was calculated in days. Cases lacking either the date or implausible values were excluded from the analysis.

### Statistical analysis and signal detection

PTs with at least three or more reports were eligible for disproportionality analysis. Four validated algorithms were applied: 1) reporting odds ratio (ROR) [13], 2) proportional reporting ratio (PRR) [14], 3) Bayesian Confidence Propagation Neural Network (BCPNN) [10], and 4) Empirical Bayesian Geometric Mean (EBGM) [15].

Positive signal criteria were defined as follows: 1) ROR: lower 95% confidence interval > 1; 2) PRR: PRR ≥ 2 with χ^2^ ≥ 4; 3) BCPNN: information component (IC) − 2 SD > 0; and 4) EBGM: EB05 > 2.

Only PT-level signals satisfying all four criteria were considered statistically significant [16]. The two-by-two contingency table and the corresponding formulas are summarized in Table S1, and the algorithm-specific thresholds are listed in Table S2.

All analyses were conducted using SAS 9.4 (SAS Institute Inc., Cary, NC, USA), GraphPad Prism 8.0 (GraphPad Software, CA, USA), and Microsoft Excel.

### Ethics approval

This study used publicly available anonymized data from the FAERS database and therefore did not require institutional ethics approval.

## Results

### Characteristics of AE reports

**Fig. 1:**
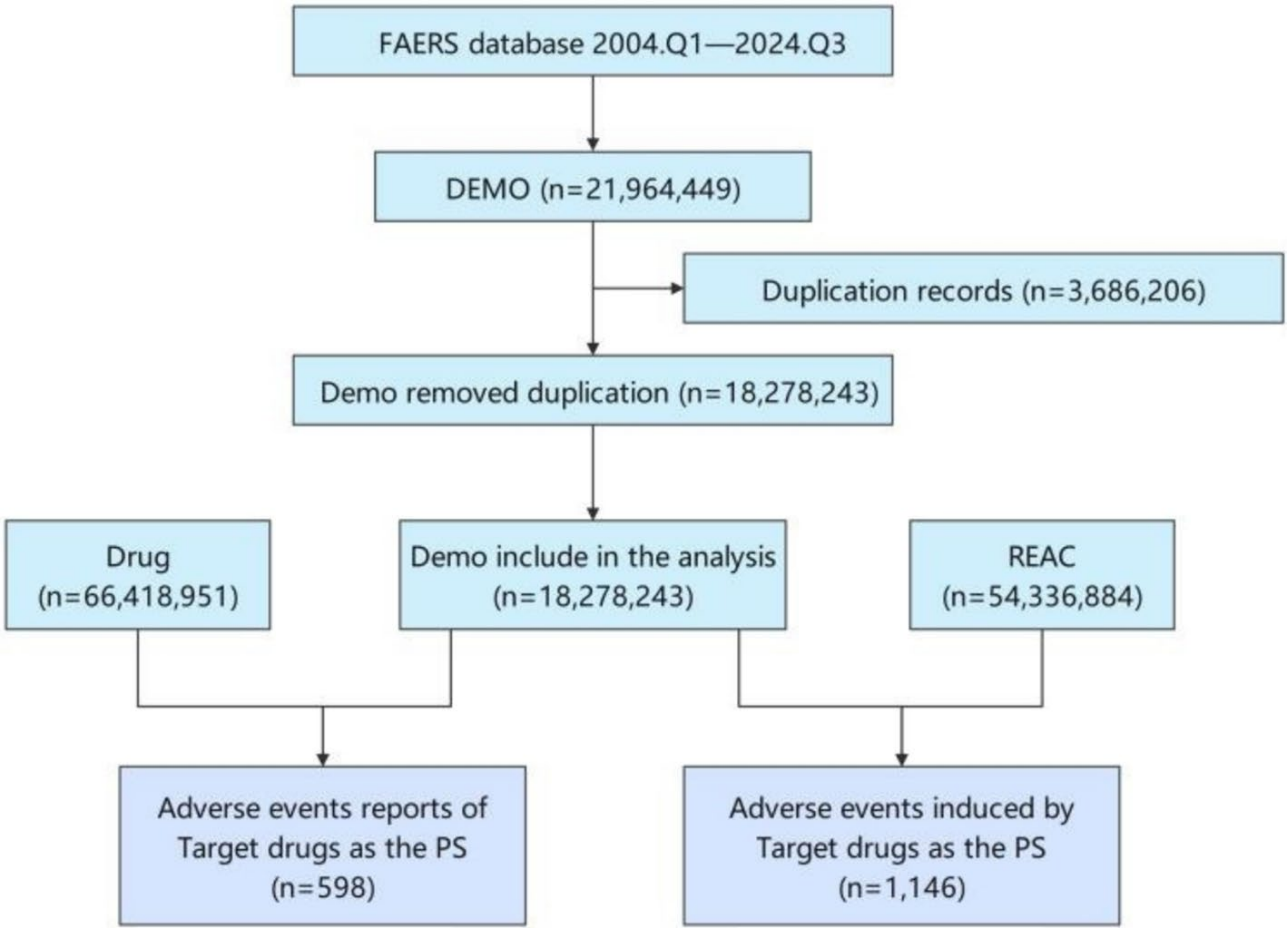
Flowchart of data extraction and processing from the Food and Drug Administration Adverse Event Reporting System (FAERS) database (2004 Q1–2024 Q3). FAERS, Food and Drug Administration Adverse Event Reporting System; DEMO, demographic file; DRUG, drug file; REAC, adverse reaction file; PS, primary suspect

A total of 598 AE reports listing casimersen as the primary suspected drug were identified in the FAERS database. Of these, 0.3% involved female patients and 98.5% involved male patients. Most cases occurred in individuals younger than 18 years (62.0%). Pharmacists were primary reporters (84. 8%), and nearly all reports (99.5%) originated from the United States. A schematic overview of the data selection and processing is shown in Fig. 1.

The annual distribution of reports showed that 40.47% of reports were recorded in 2023, and 34.45% were reported between the first and third quarters of 2024, with a higher proportion of reports observed in recent years. Detailed demographic and clinical characteristics are summarized in **Table S3**.

### Adverse events by system organ class

**Fig. 2:**
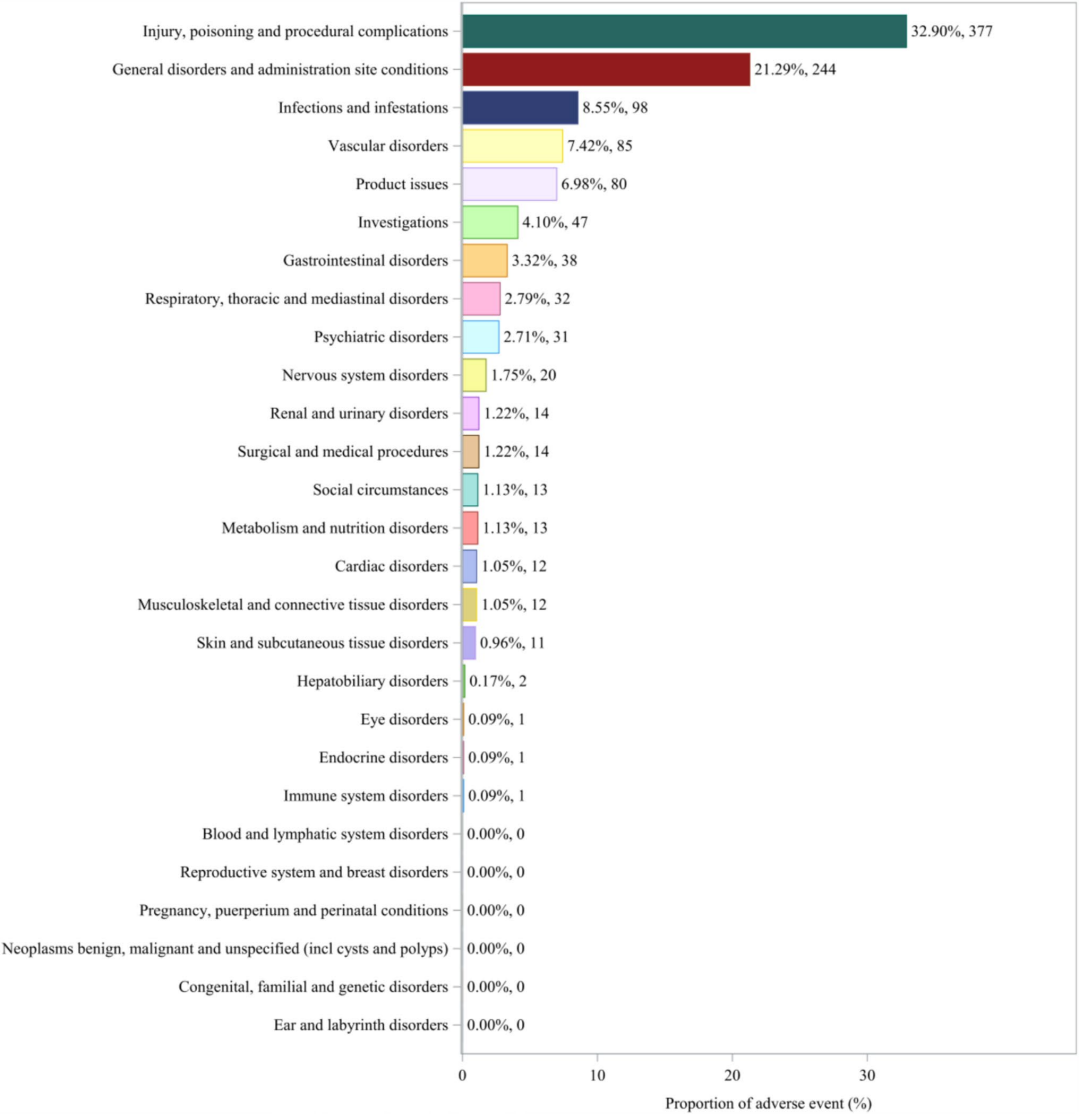
Distribution of reported System Organ Classes among adverse event reports listing casimersen as the primary suspect drug, derived from the Food and Drug Administration Adverse Event Reporting System (2004 Q1–2024 Q3)

A total of 21 SOCs were identified among the AE reports, listing casimersen as the primary suspect drug. Figure 2 illustrates the distribution of reported SOCs among the casimersen-related AE reports, and Table 1 summarizes the corresponding disproportionality results.

**Table 1:** Signal strength of casimersen-related adverse events across System Organ Classes in the United States Food and Drug Administration Adverse Event Reporting System

System Organ Class (SOC)	Number of cases	ROR (95% CI)	χ^2^	IC (IC₀₂₅)	EBGM (EBGM₀₅)
Injury, poisoning and procedural complications*	377	4.26 (3.76–4.81)	629.91	1.67 (1.49)	3.18 (2.81)
General disorders and administration site conditions	244	1.28 (1.11–1.48)	11.82	0.29 (0.08)	1.22 (1.06)
Infections and infestations	98	1.69 (1.38–2.08)	25.43	0.71 (0.40)	1.63 (1.33)
Vascular disorders*	85	3.67 (2.94–4.58)	152.86	1.80 (1.43)	3.47 (2.78)
Product issues*	80	4.58 (3.65–5.75)	208.19	2.11 (1.72)	4.33 (3.45)
Investigations	47	0.65 (0.49–0.87)	8.33	− 0.58 (− 1.00)	0.67 (0.50)
Gastrointestinal disorders	38	0.37 (0.27–0.51)	39.67	− 1.36 (− 1.81)	0.39 (0.28)
Respiratory, thoracic and mediastinal disorders	32	0.58 (0.41–0.83)	9.35	− 0.75 (− 1.25)	0.59 (0.42)
Psychiatric disorders	31	0.46 (0.32–0.66)	18.67	− 1.06 (− 1.56)	0.48 (0.33)
Nervous system disorders	20	0.19 (0.12–0.30)	67.31	− 2.29 (− 2.87)	0.21 (0.13)
Surgical and medical procedures	14	0.91 (0.54–1.54)	0.13	− 0.14 (− 0.88)	0.91 (0.54)
Renal and urinary disorders	14	0.64 (0.38–1.08)	2.89	− 0.64 (− 1.36)	0.64 (0.38)
Metabolism and nutrition disorders	13	0.52 (0.30–0.89)	5.80	− 0.94 (− 1.66)	0.52 (0.30)
Social circumstances*	13	2.46 (1.42–4.25)	11.12	1.29 (0.37)	2.44 (1.41)
Musculoskeletal and connective tissue disorders	12	0.19 (0.11–0.34)	39.84	− 2.31 (− 3.02)	0.20 (0.11)
Cardiac disorders	12	0.39 (0.22–0.69)	11.32	− 1.33 (− 2.07)	0.40 (0.22)
Skin and subcutaneous tissue disorders	11	0.17 (0.09–0.31)	43.97	− 2.49 (− 3.22)	0.18 (0.10)
Hepatobiliary disorders	2	0.19 (0.05–0.76)	6.96	− 2.39 (− 3.61)	0.19 (0.05)
Immune system disorders	1	0.08 (0.01–0.56)	10.79	− 3.65 (− 4.81)	0.08 (0.01)
Endocrine disorders	1	0.34 (0.05–2.44)	1.25	− 1.54 (− 3.01)	0.34 (0.05)
Eye disorders	1	0.04 (0.01–0.31)	21.28	− 4.51 (− 5.62)	0.04 (0.01)

Asterisks (*) indicate significant signals detected in all four algorithms. Signals were considered positive when all the following criteria were met: PRR ≥ 2 and χ^2^ > 4, lower limit of 95% confidence interval (CI) of ROR > 1, IC₀₂₅ > 0, and EBGM₀₅ > 2

Abbreviations: ROR, reporting odds ratio; PRR, proportional reporting ratio; IC, information component; EBGM, empirical Bayesian geometric mean; CI, confidence interval; IC₀₂₅ and EBGM₀₅, lower limits of the 95% confidence interval for IC and EBGM, respectively

At the SOC level, disproportionality signals meeting all four statistical criteria were observed across multiple reporting categories including injury, poisoning, procedural complications (SOC: 10,022,117; n = 377), vascular disorders (SOC: 10,047,065; n = 85), product issues (SOC: 10,077,536; n = 80), and social circumstances (SOC: 10,041,244; n = 13). These findings indicate that reports related to procedural complications and vascular disorders accounted for a substantial proportion of SOC-level reports in the FAERS database.

### Distribution of casimersen PT signals across algorithms

**Fig. 3:**
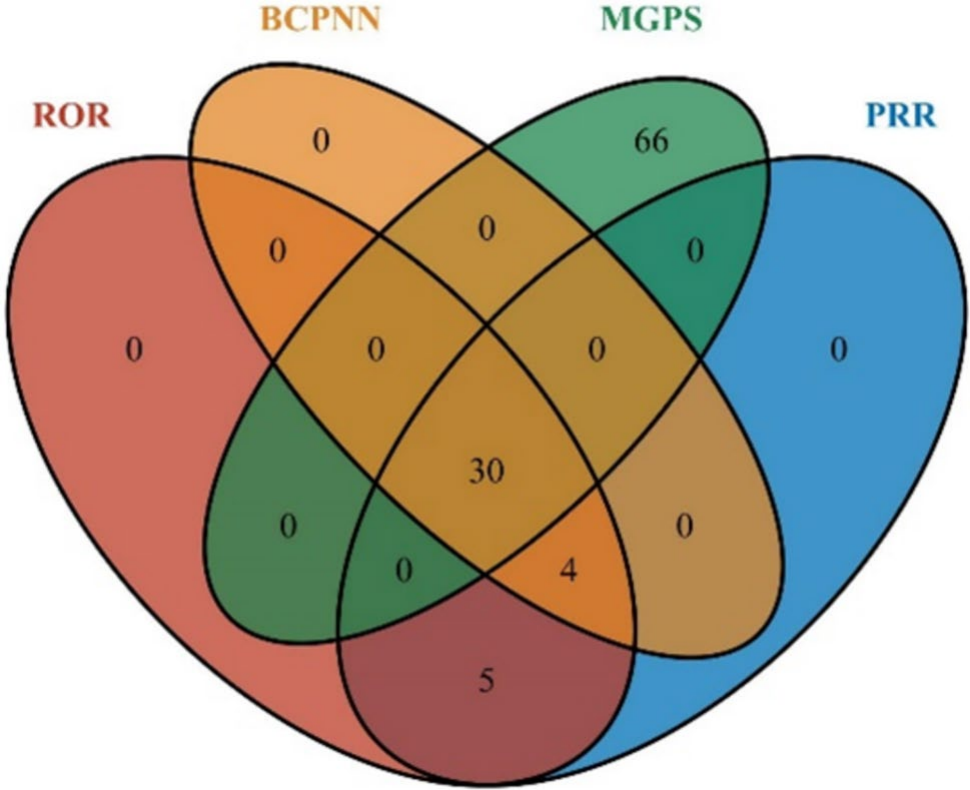
Overlap of PT-level disproportionality signals for casimersen detected using four algorithms: Reporting Odds Ratio (ROR), Proportional Reporting Ratio (PRR), Bayesian Confidence Propagation Neural Network (BCPNN), and Multi-item Gamma Poisson Shrinker (MGPS)

Four independent algorithms, ROR, PRR, BCPNN, and EBGM—105 PT-level disproportionality signals were identified. The number of effective signals detected by each method was 39, 39, 34, and 96, respectively. A visual comparison of the algorithm outputs is shown in Fig. 3. The convergence of multiple signal-detection approaches demonstrates consistency across the algorithms.

### Significant preferred terms (PTs)

Among all identified PTs, 30 PT-level disproportionality findings met the predefined statistical criteria across all four algorithms (Table 2). Commonly reported events include upper respiratory tract infection, acute respiratory failure, and influenza. In addition, several potentially novel or unlabelled PTs have been detected, including product dose omission, poor venous access, proteinuria, hematuria, chromaturia, underdose, illness, and infusion site extravasation. These PTs encompassed heterogeneous reporting categories, including clinically oriented terms, administration-related challenges, medication use processes, and non-specific descriptors. These findings describe post-marketing AE reporting patterns that warrant further evaluation in future studies.

**Table 2:** Signal strength of reports associated with casimersen ranked by empirical Bayesian geometric mean (EBGM) at the Preferred Term level in the United States Food and Drug Administration Adverse Event Reporting System

System Organ Class (SOC)	Preferred Term (PT)	Number of cases	ROR (95% CI)	χ^2^	IC (IC₀₂₅)	EBGM (EBGM₀₅)	FDA label listed (Yes/No)
Vascular disorders	Poor venous access	80	472.49 (376.09–593.59)	34,690.0	8.77 (5.76)	435.54 (346.68)	No
Social circumstances	Patient uncooperative	3	434.79 (139.30–1357.12)	1,283.25	8.75 (0.54)	429.74 (137.68)	No
Injury, poisoning and procedural complications	Exposure to SARS-CoV-2	12	357.56 (202.03–632.83)	4,190.86	8.46 (2.84)	351.22 (198.45)	No
Psychiatric disorders	Procedural anxiety	3	248.45 (79.78–773.78)	733.60	7.95 (0.53)	246.52 (79.16)	No
General disorders and administration site conditions	Catheter site swelling	3	188.64 (60.61–587.08)	556.26	7.55 (0.53)	187.41 (60.22)	No
Injury, poisoning and procedural complications	Intentional dose omission	64	183.79 (142.77–236.61)	10,945.3	7.43 (5.20)	172.96 (134.35)	No
General disorders and administration site conditions	Catheter site pain	6	138.15 (61.86–308.51)	810.31	7.10 (1.65)	137.04 (61.36)	No
Product issues	Product distribution issue	14	133.67 (78.85–226.59)	1,815.84	7.04 (3.01)	131.68 (77.68)	No
Surgical and medical procedures	Central venous catheterisation	4	71.17 (26.65–190.09)	275.36	6.15 (0.95)	70.82 (26.51)	No
General disorders and administration site conditions	Infusion site extravasation	7	60.50 (28.76–127.26)	406.60	5.91 (1.82)	60.06 (28.55)	No
General disorders and administration site conditions	No adverse event	143	53.30 (44.73–63.52)	6,417.15	5.55 (4.89)	46.73 (39.22)	No
Social circumstances	Refusal of treatment by patient	5	39.48 (16.39–95.06)	186.53	5.30 (1.23)	39.28 (16.31)	No
Product issues	Device issue	49	34.33 (25.78–45.70)	1,516.53	5.04 (3.91)	32.88 (24.69)	No
Investigations	Protein urine present	3	33.23 (10.70–103.24)	93.48	5.05 (0.43)	33.13 (10.66)	No
Injury, poisoning and procedural complications	Product dose omission issue	225	30.98 (26.78–35.84)	5,242.92	4.65 (4.29)	25.08 (21.68)	No
Injury, poisoning and procedural complications	Product communication issue	3	24.67 (7.94–76.62)	67.91	4.62 (0.39)	24.59 (7.92)	No
Social circumstances	Insurance issue	4	20.31 (7.61–54.23)	73.16	4.34 (0.77)	20.24 (7.58)	No
Product issues	Device occlusion	4	16.56 (6.20–44.21)	58.26	4.04 (0.72)	16.50 (6.18)	No
Infections and infestations	Device related infection	3	9.47 (3.05–29.40)	22.66	3.24 (0.16)	9.44 (3.04)	No
Infections and infestations	COVID-19	29	9.00 (6.22–13.01)	200.96	3.14 (2.27)	8.80 (6.08)	No
Injury, poisoning and procedural complications	Lower limb fracture	3	8.95 (2.88–27.80)	21.13	3.16 (0.14)	8.93 (2.88)	No
Renal and urinary disorders	Proteinuria	3	8.90 (2.87–27.63)	20.97	3.15 (0.13)	8.88 (2.86)	No
Respiratory, thoracic and mediastinal disorders	Acute respiratory failure	3	8.69 (2.80–26.98)	20.35	3.12 (0.13)	8.67 (2.79)	No
Product issues	Device dislocation	7	8.33 (3.96–17.51)	44.86	3.05 (1.09)	8.28 (3.94)	No
Investigations	Blood urine present	3	8.30 (2.67–25.79)	19.22	3.05 (0.11)	8.28 (2.67)	No
Renal and urinary disorders	Chromaturia	3	6.83 (2.20–21.23)	14.90	2.77 (0.03)	6.82 (2.20)	No
Injury, poisoning and procedural complications	Underdose	9	6.74 (3.50–12.99)	43.66	2.74 (1.18)	6.70 (3.47)	No
Infections and infestations	Upper respiratory tract infection	5	5.92 (2.46–14.24)	20.33	2.56 (0.52)	5.89 (2.45)	Yes
General disorders and administration site conditions	Illness	8	5.45 (2.72–10.93)	28.88	2.44 (0.90)	5.42 (2.70)	No
Infections and infestations	Influenza	10	5.14 (2.76–9.57)	33.01	2.35 (1.02)	5.10 (2.74)	Yes

FDA label listed indicates whether the preferred term is explicitly mentioned in the Adverse Reactions section of the U.S. FDA prescribing information for casimersen. This designation is descriptive only and does not imply causality, incidence, or clinical risk

PRR, proportional reporting ratio; EBGM, empirical Bayesian geometric mean; EBGM₀₅, lower limit of the 95% confidence interval of EBGM; IC, information component; IC₀₂₅, lower limit of the 95% confidence interval of IC; CI, confidence interval; PT, preferred term

### Subgroup analysis

Subgroup evaluations according to age revealed differences in the reported PT distributions. In patients younger than 18 years of age, the specific PTs that met the positive signal criteria were dehydration, infusion site extravasation, illness, and influenza. In contrast, malaise and pneumonia were exclusively observed in patients aged 18 years. The age-related distribution of PTs is summarized in **Tables S4** and **S5**, which describe age-stratified reporting patterns in the FAERS database.

### Time-to-onset of adverse events

**Fig. 4:**
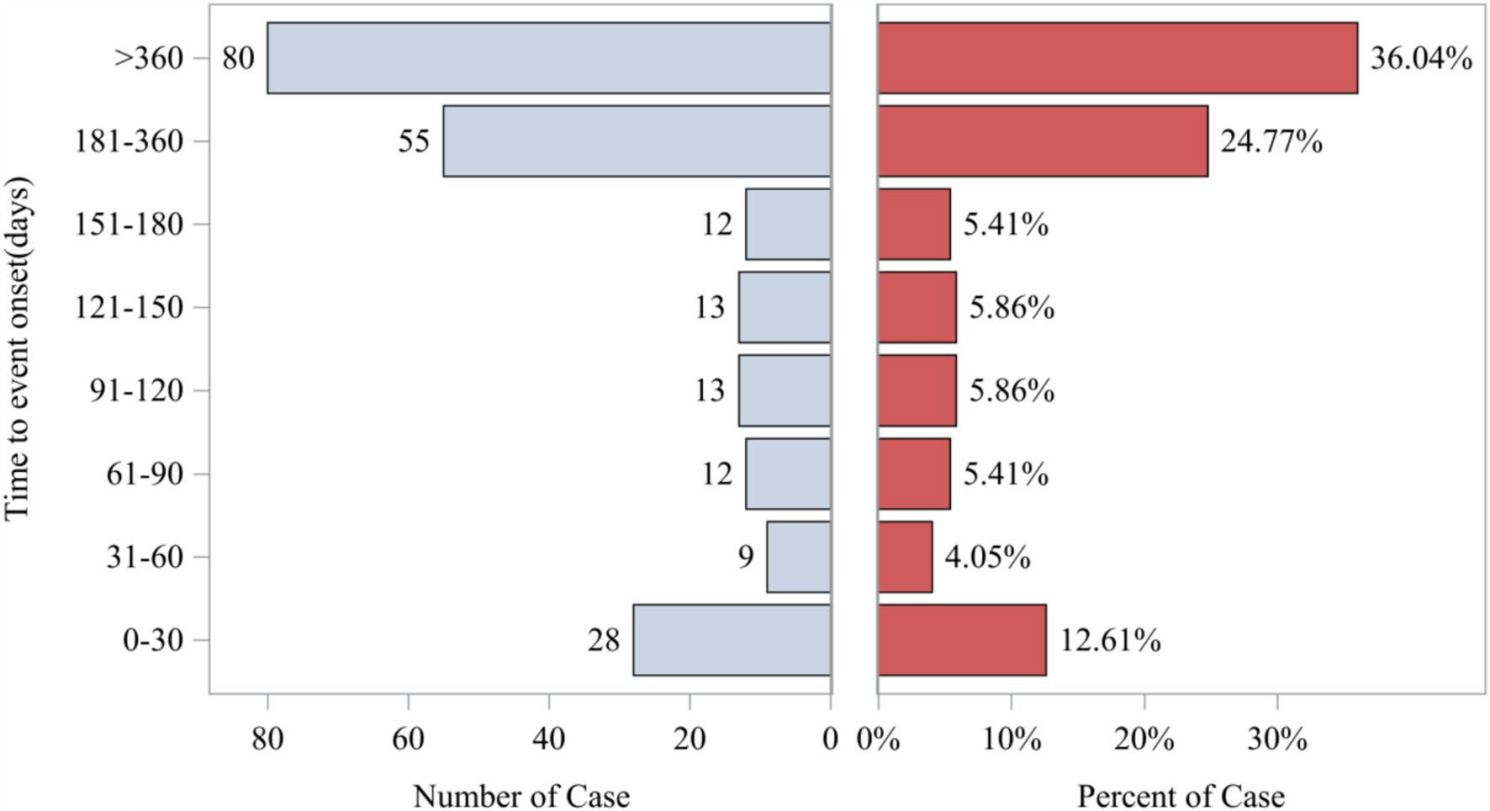
Distribution of reported time to onset of adverse events for casimersen in the FAERS database

Among the 598 cases, 222 contained complete and valid onset data. The mean time-to-onset was 315 days, with a median of 253 days (interquartile range [IQR] 101–490 days). Approximately 36.04% of all AEs occurred within one year of initiating casimersen therapy (n = 80). The distribution of the onset intervals is shown in Fig. 4 and the cumulative incidence of AEs is shown in Figure S1. These results indicated that many reported AEs occurred after several months of treatment exposure, reflecting a delayed temporal distribution of reports within the FAERS database.

## Discussion

### Principal findings

This pharmacovigilance study provides a descriptive analysis of postmarketing AE reporting for casimersen using real-world FAERS data. A total of 598 AE reports were identified, most of which involved male (98.5%) and pediatric (< 18 years, 62.0%) patients, consistent with the epidemiology of DMD.

The disproportionality analysis identified 21 affected organ systems, with the most prominent being injury, poisoning, procedural complications, vascular disorders, product issues, and social circumstances. Among the 30 PTs that met all four algorithm criteria, both previously reported and less-frequently described preferred terms were identified. Common events such as upper respiratory tract infections, acute respiratory failure, and influenza corresponded to prior clinical trial findings, whereas additional PTs, including proteinuria, hematuria, chromaturia, infusion site extravasation, poor venous access, underdose, and product dose omission, were identified, warranting further evaluation in future studies.

### Clinical interpretation and pharmacist implications

Renal-related AE reports were identified in the FAERS database, suggesting that renal-related reporting was observed in the post-marketing FAERS data for casimersen. Periodic evaluation of glomerular filtration rate, urine dipstick results, serum cystatin C, and protein-to-creatinine ratio has been described in the literature as part of renal safety assessment for antisense oligonucleotide therapies [9].

Procedural and product-related complications, such as poor venous access and infusion site extravasation, likely reflect the challenges of repeated intravenous infusions in patients with DMD who often experience muscle wasting and increased subcutaneous fat deposition. These observations provide contextual insight into the procedural challenges associated with repeated intravenous administration, which may help contextualize infusion-related reporting patterns in routine practice. Additionally, reports coded under preferred terms such as “dose omission” and “underdose” may reflect adherence and psychosocial factors in pediatric and adolescent patients, which merit attention in multidisciplinary care settings.

### Age-specific differences

Subgroup analyses showed that certain preferred terms, including dehydration, infusion site extravasation, illness, and influenza, were reported more frequently in patients aged < 18 years, whereas malaise and pneumonia were reported predominantly in adults. These differences may stem from developmental physiology, varying immune responses, or cumulative drug exposure. The susceptibility of younger patients to dehydration or infection could be related to reduced swallowing capacity and weaker immune defenses, whereas adults may experience systemic fatigue or respiratory complications due to disease progression. These findings suggest age-related differences in the reported AE patterns that warrant further investigation.

### Time-to-onset and exposure duration

Among 222 evaluable cases, the median AE onset was 253 days (IQR, 101–490 days), and 36% occurred within the first year of treatment. The observed distribution reflected a delayed temporal pattern of reported events in the FAERS database, with a substantial proportion of reports occurring after extended treatment exposure.

### Comparison with existing evidence

The AE reporting patterns here align with that observed for other exon-skipping ASOs, such as eteplirsen and golodirsen, which also exhibit renal excretion and potential infusion-related complications [9, 17, 18]. The absence of significant disproportionality signals for common AEs (e.g., headache, cough, fever, arthralgia) likely reflects the high background reporting rates in FAERS, which can dilute drug-specific signal strength [19, 20]. Therefore, the absence of disproportionality should not be interpreted as an absence of an association in spontaneous reporting. This consistency across ASO drugs suggests a potential class-related reporting pattern, with renal and infusion-site events being particularly noteworthy.

### Strengths and limitations

A major strength of this study is the multi-algorithmic disproportionality approach, which enhances the consistency of the PT-level findings across methods and minimizes spurious associations. Additional strengths include standardized MedDRA coding, inclusion of time-to-onset analysis, and subgroup evaluation, all of which contribute to clinical interpretability.

However, this study has several limitations. FAERS data are subject to underreporting, reporting bias, and missing information, which may limit the quantitative assessment. Stimulated reporting, indication bias, and confounding by disease progression or treatment-related factors may also influence the reporting patterns. Reported AEs do not confirm causality between casimersen and the event [21]. In addition, disproportionality signals reflect disproportionate reporting rather than incidence, clinical risk, or confirmed adverse drug reactions, and have no inherent clinical meaning. All disproportionality analyses were conducted at the MedDRA-preferred term level. Although PT-level analyses are standard in FAERS-based pharmacovigilance, they may be overly granular, and some PTs represent heterogeneous or non-clinical reporting categories. Higher-level aggregation approaches (e.g., high-level terms or standardized MedDRA queries) may improve clinical interpretability and warrant exploration in future studies. Unmeasured confounders, such as concomitant medications or comorbidities, may influence AE patterns [11]. Furthermore, the incidence rates could not be estimated because of the lack of denominator data, and comparative analyses with other DMD therapies were not conducted. These factors should be considered when interpreting the findings of this study.

Future studies involving real-world registries and head-to-head comparisons among ASO therapies are needed to evaluate whether these reporting signals correspond to clinically meaningful associations and clarify their clinical context.

### Clinical and research implications

This study describes the post-marketing AE reporting patterns of casimersen in a real-world setting. In routine practice, attention to renal status, infusion-site procedures, and adherence challenges may be relevant, particularly in younger patients. Routine pharmacovigilance, early AE recognition, and coordinated care may help contextualize and interpret AE reports during long-term therapy.

From a research perspective, these findings highlight the value of FAERS as a postmarketing surveillance tool for rare disease therapeutics. Expanding collaborative pharmacovigilance networks and establishing longitudinal registries could further refine the safety monitoring of antisense oligonucleotide drugs.

## Conclusion

This study describes the post-marketing adverse event reporting patterns associated with casimersen using the FAERS database. Most of the reported PTs were consistent with the known safety profile; however, additional PT-level disproportionality findings were identified, including product dose omission, poor venous access, proteinuria, hematuria, chromaturia, underdosing, and infusion site extravasation. The time-to-onset analysis showed that many reported events occurred after prolonged treatment exposure, reflecting a delayed reporting pattern over time. These findings describe reporting patterns that may merit further evaluation, particularly with respect to renal events, infusion-site reporting, and adherence-related reporting. Given the inherent limitations of spontaneous reporting systems and the exploratory nature of disproportionality analyses, further prospective and long-term real-world studies are required to evaluate the clinical relevance of these reporting patterns.

## Supplementary Information

Below is the link to the electronic supplementary material.

Supplementary file1 (DOCX 97 kb)

## Data Availability

The datasets generated and analyzed in the current study are available from the corresponding author upon reasonable request.

## References

[1] Duan D, Goemans N, Takeda S, et al. Duchenne muscular dystrophy. Nat Rev Dis Primers. 2021;7(1):13. 10.1038/s41572-021-00248-3. 33602943 10.1038/s41572-021-00248-3 PMC10557455

[2] Verhaart IEC, Aartsma-Rus A. Therapeutic developments for Duchenne muscular dystrophy. Nat Rev Neurol. 2019;15(7):373–86. 10.1038/s41582-019-0203-3. 31147635 10.1038/s41582-019-0203-3

[3] Larson CM, Henderson RC. Bone mineral density and fractures in boys with Duchenne muscular dystrophy. J Pediatr Orthop. 2000;20(1):71–4. 10641693

[4] Catalano A, Vita GL, Bellone F, et al. Bone health in Duchenne muscular dystrophy: clinical and biochemical correlates. J Endocrinol Invest. 2022;45(3):517–25. 10.1007/s40618-021-01676-4. 34524678 10.1007/s40618-021-01676-4

[5] Chang M, Cai Y, Gao Z, et al. Duchenne muscular dystrophy: pathogenesis and promising therapies. J Neurol. 2023;270(8):3733–49. 10.1007/s00415-023-11796-x. 37258941 10.1007/s00415-023-11796-x

[6] Roberts TC, Langer R, Wood MJA. Advances in oligonucleotide drug delivery. Nat Rev Drug Discov. 2020;19(10):673–94. 10.1038/s41573-020-0075-7. 32782413 10.1038/s41573-020-0075-7 PMC7419031

[7] Bennett CF. Therapeutic antisense oligonucleotides are coming of age. Annu Rev Med. 2019;70:307–21. 10.1146/annurev-med-041217-010829. 30691367 10.1146/annurev-med-041217-010829

[8] Wagner KR, Kuntz NL, Koenig E, et al. Safety, tolerability, and pharmacokinetics of casimersen in patients with Duchenne muscular dystrophy amenable to exon 45 skipping: a randomized, double-blind, placebo-controlled, dose-titration trial. Muscle Nerve. 2021;64(3):285–92. 10.1002/mus.27347. 34105177 10.1002/mus.27347 PMC9290993

[9] Wu H, Wahane A, Alhamadani F, et al. Nephrotoxicity of marketed antisense oligonucleotide drugs. Curr Opin Toxicol. 2022. 10.1016/j.cotox.2022.100373. 37193356 10.1016/j.cotox.2022.100373 PMC10174585

[10] Shu Y, Ding Y, Liu Y, et al. Post-marketing safety concerns with secukinumab: a disproportionality analysis of the FDA adverse event reporting system. Front Pharmacol. 2022;13:862508. 10.3389/fphar.2022.862508. 35754494 10.3389/fphar.2022.862508 PMC9214234

[11] Gliklich R, Dreyer N, Leavy M, eds. Registries for Evaluating Patient Outcomes: A User's Guide. Third edition. Two volumes. (Prepared by the Outcome DEcIDE Center [Outcome Sciences, Inc., a Quintiles company] under Contract No. 290 2005 00351 TO7.) AHRQ Publication No. 13(14)-EHC111. Rockville, MD: Agency for Healthcare Research and Quality. April 2014. http://www.effectivehealthcare.ahrq.gov/registries-guide-3.cfm..

[12] Brown EG. Using MedDRA: implications for risk management. Drug Saf. 2004;27(8):591–602. 10.2165/00002018-200427080-00010. 15154830 10.2165/00002018-200427080-00010

[13] Müller A, Edmonston TB, Corao DA, et al. Exclusion of breast cancer as an integral tumor of hereditary nonpolyposis colorectal cancer. Cancer Res. 2002;62(4):1014–9. 11861375

[14] Evans SJ, Waller PC, Davis S. Use of proportional reporting ratios (PRRs) for signal generation from spontaneous adverse drug reaction reports. Pharmacoepidemiol Drug Saf. 2001;10(6):483–6. 10.1002/pds.677. 11828828 10.1002/pds.677

[15] Szarfman A, Machado SG, O’Neill RT. Use of screening algorithms and computer systems to efficiently signal higher-than-expected combinations of drugs and events in the US FDA’s spontaneous reports database. Drug Saf. 2002;25(6):381–92. 10.2165/00002018-200225060-00001. 12071774 10.2165/00002018-200225060-00001

[16] Shu Y, Ding Y, He X et al.. Hematological toxicities in PARP inhibitors: a real-world study using FDA adverse event reporting system (FAERS) database. Cancer Med. 2023;12(3):3365–75. 10.1002/cam4.5062. 35871395 10.1002/cam4.5062 PMC9939145

[17] Alhamadani F, Zhang K, Parikh R et al. Adverse drug reactions and toxicity of the Food and Drug Administration-approved antisense oligonucleotide drugs. Drug Metab Dispos. 2022;50(6):879–87. 10.1124/dmd.121.000418. 35221289 10.1124/dmd.121.000418 PMC11022857

[18] Kurreck J. Antisense technologies. Improvement through novel chemical modifications. Eur J Biochem. 2003;270(8):1628–44. 10.1046/j.1432-1033.2003.03555.x. 12694176 10.1046/j.1432-1033.2003.03555.x

[19] Sakaeda T, Tamon A, Kadoyama K, et al.. Data mining of the public version of the FDA adverse event reporting system. Int J Med Sci. 2013;10(7):796–803. 10.7150/ijms.6048. 23794943 10.7150/ijms.6048 PMC3689877

[20] Stephenson WP, Hauben M. Data mining for signals in spontaneous reporting databases: proceed with caution. Pharmacoepidemiol Drug Saf. 2007;16(4):359–65. 10.1002/pds.1323. 17019675 10.1002/pds.1323

[21] Maciá-Martínez MA, de Abajo FJ, Roberts G et al. An empirical approach to explore the relationship between measures of disproportionate reporting and relative risks from analytical studies. Drug Saf. 2016;39(1):29–43. 10.1007/s40264-015-0351-3. 26507885 10.1007/s40264-015-0351-3

