# Supplementary material for: Analysis of adverse event reporting with casimersen: a pharmacovigilance study based on the United States food and drug administration adverse event reporting system database
Source: Int J Clin Pharm. 2026 Feb 26;48(3):1048–57. doi: 10.1007/s11096-026-02103-5 (PMC13176065; doi:10.1007/s11096-026-02103-5)
Supplement: Supplementary file 1 — Supplementary file1 (DOCX 97 kb) [file 11096_2026_2103_MOESM1_ESM.docx]

**Supplementary Information**

**Introduction**

We prepared the supplementary materials because the journal limits the number of figures and tables that can be included in the main manuscript to a maximum of six. To comply with this restriction while ensuring that all essential methodological details and supporting results are fully available to readers, the remaining figures and tables have been organized and placed in the supplementary section. This approach allows us to present the complete dataset and analytical outputs without exceeding the journal’s formatting requirements.

**Figure S1**. Cumulative incidence curve of adverse events associated with casimersen.

**
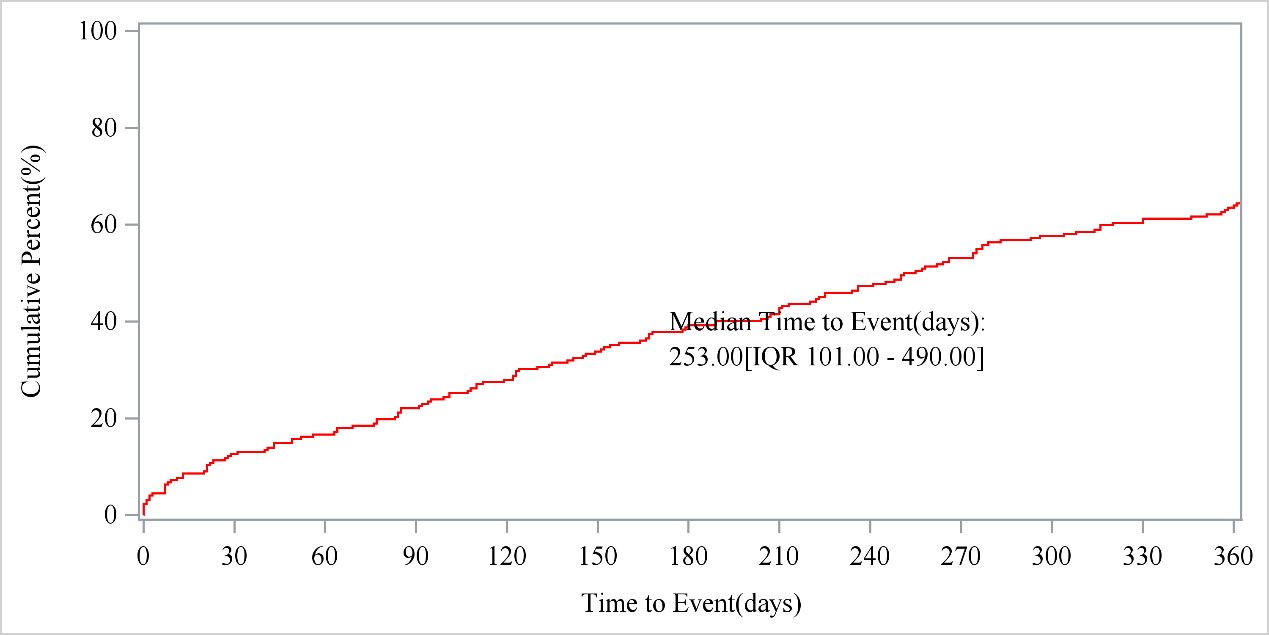
**

**Table S1.** Two-by-two contingency table for disproportionality analyses

| **Item** | **Target PTs** | **Non-Target PTs** | **Total** |
| --- | --- | --- | --- |
| **Target drug** | a | b | a+b |
| **Other drugs** | c | d | c+d |
| **Total** | a+c | b+d | a+b+c+d |

Note: a = number of reports containing both the target drug and the target preferred term. b = number of reports containing the target drug but not the target preferred term. c = number of reports containing the target preferred term with other drugs. d = number of reports containing neither the target drug nor the target preferred term.

**Table S2.** Four primary algorithms used for signal detection

| **Methods** | **Formula** | **Signal standard** |
| --- | --- | --- |
| **ROR** | 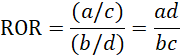  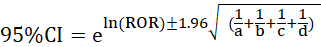 | 95%CI > 1, a≥3 |
| **PRR** | 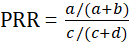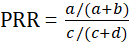  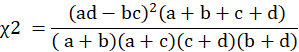 | PRR≥2, χ²≥4, a≥3 |
| **BCPNN** | IC=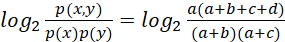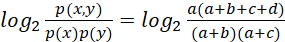  E(IC)=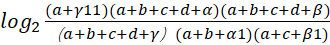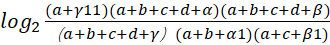  V(IC)=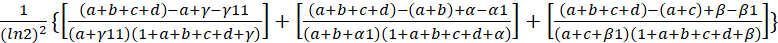 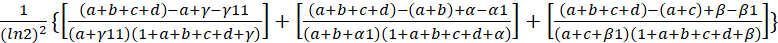  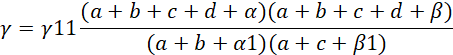  IC025=E(IC)-2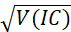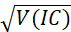 | IC025 > 0 |
| **MGPS** | 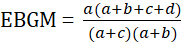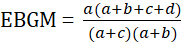  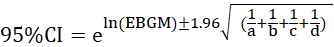 | EBGM05 > 2 |

ROR, reporting odds ratio; PRR, proportional reporting ratio; BCPNN, Bayesian confidence propagation neural network; MGPS, multi-item gamma Poisson shrinker; EBGM, empirical Bayesian geometric mean; CI, confidence interval; χ², chi-squared; IC, information component; IC₀₂₅, lower limit of the 95% one-sided confidence interval of the information component; EBGM₀₅, lower 95% one-sided confidence limit of the empirical Bayesian geometric mean.

**Table S3.** Demographic and clinical characteristics of reports associated with casimersen from the United States Food and Drug Administration Adverse Event Reporting System (first quarter of 2004 to third quarter of 2024)

| Characteristic | Category | Number of cases (n = 598) | Proportion (%) |
| --- | --- | --- | --- |
| Sex | Female | 2 | 0.33 |
|  | Male | 589 | 98.49 |
|  | Not specified | 7 | 1.17 |
| Age (years) | < 18 | 371 | 62.04 |
|  | 18–44 | 125 | 20.90 |
|  | 45–64 | 1 | 0.17 |
|  | ≥ 65 | 0 | 0 |
|  | Not specified | 101 | 16.89 |
| Reporter type | Consumer | 83 | 13.88 |
|  | Physician | 7 | 1.17 |
|  | Pharmacist | 507 | 84.78 |
|  | Not specified | 1 | 0.17 |
| Top reporting countries | United States | 595 | 99.50 |
|  | United Kingdom | 1 | 0.17 |
| Reporting year | 2021 | 17 | 2.84 |
|  | 2022 | 133 | 22.24 |
|  | 2023 | 242 | 40.47 |
|  | 2024 (Q1–Q3) | 206 | 34.45 |
| Reported outcome | Life-threatening | 3 | 0.50 |
|  | Hospitalization – initial or prolonged | 66 | 11.04 |
|  | Death | 3 | 0.50 |
|  | Other outcomes | 23 | 3.85 |

Note: Percentages are calculated based on the total number of casimersen-related reports (n = 598). Minor discrepancies in percentages are due to rounding.
FAERS, Food and Drug Administration Adverse Event Reporting System.

**Table S4.** All adverse events meeting the positive signal criteria at the Preferred Term level for casimersen in patients under 18 years of age, as reported in the United States Food and Drug Administration Adverse Event Reporting System

| Preferred Term (PT) | Number of cases | ROR (95% CI) | χ² | IC (IC₀₂₅) | EBGM (EBGM₀₅) |
| --- | --- | --- | --- | --- | --- |
| Product dose omission issue | 173 | 48.69 (41.03–57.79) | 6,101.68 | 5.21 (4.69) | 37.01 (31.18) |
| No adverse event | 116 | 41.74 (34.20–50.95) | 3,837.94 | 5.12 (4.47) | 34.90 (28.59) |
| Poor venous access | 53 | 286.28 (213.84–383.26) | 12,750.88 | 7.92 (5.05) | 242.42 (181.08) |
| Intentional dose omission | 50 | 226.69 (168.41–305.15) | 9,724.82 | 7.62 (4.91) | 196.35 (145.87) |
| Device issue | 38 | 24.55 (17.69–34.07) | 807.30 | 4.53 (3.41) | 23.15 (16.68) |
| COVID-19 | 13 | 13.74 (7.93–23.81) | 150.13 | 3.75 (2.05) | 13.45 (7.76) |
| Product distribution issue | 9 | 177.56 (90.15–349.69) | 1,467.13 | 7.37 (2.30) | 164.94 (83.75) |
| Exposure to SARS-CoV-2 | 9 | 357.66 (177.99–718.70) | 2,801.70 | 8.29 (2.31) | 313.17 (155.85) |
| Dehydration | 8 | 6.32 (3.14–12.69) | 35.32 | 2.64 (1.01) | 6.25 (3.11) |
| Underdose | 8 | 8.01 (3.99–16.09) | 48.40 | 2.98 (1.19) | 7.91 (3.94) |
| Influenza | 7 | 5.56 (2.64–11.72) | 25.90 | 2.46 (0.79) | 5.51 (2.62) |
| Device dislocation | 6 | 18.24 (8.15–40.84) | 96.33 | 4.17 (1.29) | 17.99 (8.03) |
| Illness | 6 | 10.53 (4.71–23.54) | 51.11 | 3.38 (1.05) | 10.41 (4.66) |
| Infusion site extravasation | 5 | 44.47 (18.33–107.92) | 207.70 | 5.44 (1.23) | 43.50 (17.92) |
| Catheter site pain | 4 | 91.33 (33.64–247.94) | 344.06 | 6.46 (0.94) | 87.97 (32.40) |
| Central venous catheterisation | 4 | 50.92 (18.89–137.27) | 191.19 | 5.64 (0.90) | 49.75 (18.46) |
| Refusal of treatment by patient | 3 | 45.98 (14.66–144.25) | 129.32 | 5.49 (0.44) | 45.06 (14.37) |
| Device occlusion | 3 | 11.94 (3.83–37.20) | 29.82 | 3.57 (0.22) | 11.85 (3.80) |
| Procedural anxiety | 3 | 394.16 (117.32–1324.34) | 1,025.25 | 8.42 (0.43) | 343.62 (102.27) |
| Patient uncooperative | 3 | 212.24 (65.44–688.33) | 583.30 | 7.62 (0.47) | 196.35 (60.54) |
| Insurance issue | 3 | 29.04 (9.29–90.79) | 80.05 | 4.84 (0.40) | 28.63 (9.16) |
| Product communication issue | 3 | 50.16 (15.98–157.50) | 141.39 | 5.62 (0.45) | 49.09 (15.63) |

Abbreviations: PRR, proportional reporting ratio; EBGM, empirical Bayesian geometric mean; EBGM₀₅, lower limit of the 95% confidence interval of EBGM; IC, information component; IC₀₂₅, lower limit of the 95% confidence interval of IC; CI, confidence interval; PT, preferred term.

**Table S5.** All adverse events meeting the positive signal criteria at the Preferred Term level for casimersen in patients over 18 years of age, as reported in the United States Food and Drug Administration Adverse Event Reporting System

| Preferred Term (PT) | Number of cases | ROR (95% CI) | χ² | IC (IC₀₂₅) | EBGM (EBGM₀₅) |
| --- | --- | --- | --- | --- | --- |
| Product dose omission issue | 46 | 32.12 (23.30–44.27) | 1,124.40 | 4.71 (3.63) | 26.23 (19.03) |
| No adverse event | 25 | 66.37 (43.86–100.41) | 1,441.98 | 5.90 (3.60) | 59.56 (39.37) |
| Poor venous access | 21 | 407.84 (260.13–639.44) | 7,701.57 | 8.53 (3.73) | 368.64 (235.12) |
| Intentional dose omission | 13 | 195.33 (111.56–342.03) | 2,366.12 | 7.52 (2.91) | 183.95 (105.05) |
| Device issue | 10 | 26.94 (14.30–50.75) | 239.37 | 4.69 (2.10) | 25.86 (13.73) |
| Malaise | 8 | 5.64 (2.79–11.41) | 29.52 | 2.46 (0.89) | 5.49 (2.71) |
| Pneumonia | 6 | 7.83 (3.48–17.60) | 34.84 | 2.94 (0.86) | 7.66 (3.41) |
| COVID-19 | 6 | 9.10 (4.05–20.47) | 42.19 | 3.15 (0.96) | 8.90 (3.96) |
| Product distribution issue | 3 | 169.77 (54.21–531.61) | 494.59 | 7.38 (0.51) | 166.84 (53.28) |

Abbreviations: PRR, proportional reporting ratio; EBGM, empirical Bayesian geometric mean; EBGM₀₅, lower limit of the 95% confidence interval of EBGM; IC, information component; IC₀₂₅, lower limit of the 95% confidence interval of IC; CI, confidence interval; PT, preferred term.
